# Reliability-based mix design for concrete compressive strength using a physics-prior residual-learning surrogate with calibrated uncertainty

**DOI:** 10.1371/journal.pone.0350575

**Published:** 2026-06-05

**Authors:** Pengfei Qu, Lei Song, Sihan Wang

**Affiliations:** School of Management Science and Engineering, Shandong Technology and Business University, Yantai, China; Jazan University College of Engineering, SAUDI ARABIA

## Abstract

An integrated framework is presented for concrete compressive strength prediction and reliability-based mix design under data-scarce conditions, in which a physics-prior residual surrogate and uncertainty decomposition are combined. A physics-consistent baseline is constructed from an effective water-to-binder ratio with age effects, time-dependent reactivity of supplementary cementitious materials and the influence of superplasticizer on effective water demand, and is globally calibrated on the training data. A small residual neural network is then superposed on this baseline with explicit regularization, so that remaining nonlinear interactions are learned while the physical scale and monotonicity are preserved. SHAP values and partial dependence curves are used to confirm the dominant positive roles of age and cementitious content, the negative effect of water, and physically plausible nonlinear effects of admixtures and aggregates. The framework is evaluated on a publicly available high-performance concrete strength dataset containing 1,030 mixtures and 8 input variables; the hybrid Physics+Data model attains *R*^2^ = 0.9252 and RMSE = 4.39 MPa on an independent test set and maintains similar accuracy when only 40% of the training samples are used. Five-fold cross-validation confirms the stability of these results. Refined uncertainty quantification is carried out by combining mild Monte Carlo dropout, feature and physics-parameter perturbations and a single scaling factor for coverage calibration, yielding nominal 95% prediction intervals with about 95.1% empirical coverage and showing the physics sub-model as the dominant source of variance. On the calibrated surrogate, 28-day reliability-based design maps in the *w*/*b*–*C* plane for a 40 MPa strength target are produced, from which mix recommendations such as w/b≈0.33–0.36 and C≈340–360 kg/m^3^ for *P* ≥ 0.80 are derived.

## 1. Introduction

Concrete is recognized as one of the most widely used materials in global infrastructure, and 28-day compressive strength has long been employed as the core indicator for mix design and quality acceptance [1,2]. Extensive empirical and microscopic experiments have demonstrated that water-to-binder ratio (w/b) exerts dominant influence on strength, with increased w/b typically resulting in elevated porosity and reduced strength [3–5]. The formation of concrete strength is rooted in hydration kinetics and pore structure evolution, and systematic summaries of mechanisms, rates, and phase transformation processes have been provided in related reviews [6]. In modern systems where mineral admixtures and chemical additives are introduced, influencing factors are numerous and nonlinear coupling is enhanced, making empirical/semi-empirical models based on limited variables difficult to generalize stably [7,8]. In recent years, machine learning (ML) has been rapidly developed in concrete strength prediction, demonstrating superior accuracy compared to traditional regression across various datasets and working conditions, and providing insights that can be used for design [9,10]. Among these developments, a public benchmark dataset containing 1030 samples and 8 input variables has been widely used for algorithm comparison, reproducible experiment construction, and visualization tool development [2,9]. However, purely data-driven models still face challenges in cross-domain extrapolation, interpretability, and physical consistency (such as monotonicity, conservation/constraints) [11]. Physics-guided/physics-informed machine learning (PIML), which explicitly injects conservation relationships, constraints, or prior structures during the training process, is considered an effective approach for enhancing trustworthiness and generalization capability [12].

Traditional empirical and physics-oriented models are widely adopted in mix design, and compressive strength relationships are typically established with mix parameters and age (or maturity) as independent variables. At the mix design level, water-to-binder ratio is emphasized as the primary control parameter, while age effects are often summarized and quantified through maturity methods [12]. At the mechanistic level, hydration kinetics and pore structure evolution provide microscopic foundations and interpretable frameworks for strength development [6]. For composite cementitious material systems, time-dependent contributions of slag and fly ash are frequently reported: strength reduction may occur at early ages, while compensatory gains exist in the medium to late stages, with magnitudes closely related to chemical composition, fineness, replacement ratio, and other factors [13,14]. Polycarboxylate-based water reducers not only reduce mixing water demand but may also alter the dissolution-nucleation-growth pathways of cement systems, thereby indirectly affecting strength evolution and final structure [15,16,17]. However, parameters in these semi-empirical formulas often lack universality across different sites and material sources, are constrained by raw material fluctuations and dataset distribution shifts, and require recalibration in new scenarios [18]. Nonlinear coupling between admixture reactivity, additive dosage, and curing conditions makes it difficult for low-order formulas to characterize completely and robustly [13,18]. Therefore, when applications exceed the original calibration domain, even though dominant monotonic trends can be maintained, physical/empirical models may still produce systematic biases. Nevertheless, the value of physical/empirical models in interpretability and constraint consistency remains prominent, and these models can provide anchor points and boundaries for more complex methods.

In recent years, purely data-driven regression and deep learning models have been extensively adopted in concrete compressive strength prediction and mix design decision support, covering various scenarios involving both laboratory data and field-compiled datasets [19–21]. On larger-scale public and specialized datasets, modern learners such as ensemble learning and gradient boosting are often reported to achieve higher accuracy and stability, maintaining competitiveness across multiple strength grades and material types [22]. For example, an improved random forest regressor applied to the same Yeh benchmark dataset achieved *R*^2^ = 0.931 and MAE  =  3.21 MPa through optimised splitting and ensemble strategies [23]; Bayesian-optimised fully connected networks have further improved prediction accuracy on similar datasets [24]; and interpretable deep neural network frameworks have recently been deployed to balance predictive power with feature-level transparency [25]. However, model sensitivity to training distributions, lack of explicit physical constraints, and performance degradation on out-of-distribution (OOD) samples are still considered important bottlenecks for engineering deployment, requiring mitigation in both methodological and validation aspects [26]. To enhance trustworthiness and obtain mechanistic insights, emphasis has been placed on examining feature attribution, monotonic trends, and interaction effects through post-hoc interpretability tools, with appropriate trade-offs being made between “interpretable modeling vs. explaining black boxes” [27,28]. At the tool level, partial dependence/accumulated dependence and their interaction visualizations can be used to examine trends and directionality of univariate and bivariate interactions, reducing understanding biases caused by model complexity [29]. For concrete data applications, methods such as SHAP have been employed to confirm dominant factors like “age, cementitious material content, and water usage,” and reveal nonlinear marginal effects of admixtures and aggregates, helping to integrate empirical rules with data evidence [19]. Meanwhile, uncertainty quantification (UQ) is regarded as a necessary condition for risk-aware and conservative design, where consensus has been formed on the distinction between “epistemic uncertainty (model/parameter/structural)” and “aleatoric uncertainty (data/noise)” [30]. Practical UQ approaches for deep learning (such as ensembles, approximate Bayesian methods, MC Dropout, and test-time perturbations) and evaluation guidelines have been systematized in reviews, providing operational recipes for engineering implementation [31]. Beyond providing intervals and probabilities, emphasis should be placed on “calibration”: nominal coverage rates need to match empirical coverage rates on validation sets, enabling uncertainty metrics to be directly used for threshold determination and reliability measurement [32,33].

Hybrid modeling has gradually been established as the primary direction for combining physics-based mechanisms with data-driven approaches. A class of design patterns is proposed in relevant research: interpretable baselines are first provided by mechanistic models, then corrections for deviation components are performed by data models, thus achieving a balance between bias and variance [34]. Within this framework, physics-informed deep learning methods are continuously developed. From early PINN where governing equation residuals are incorporated into loss functions, to subsequent improvements focused on convergence and efficiency, it is demonstrated that under small-sample and extrapolation conditions, robustness can be significantly enhanced by physical constraints [35,36]. More recently, physics-informed loss functions have been designed specifically for concrete strength prediction to embed domain constraints directly into neural network training [37]. Meanwhile, uncertainty analysis is also strengthened. Explicit decomposition of uncertainties from sources such as models, parameters, and noise is proposed by researchers, enabling prediction intervals to be combined with physical consistency, providing a basis for engineering risk control [38]. In reliability design optimization, lightweight surrogate models are gradually adopted to replace direct simulations, supplemented by multi-fidelity and local update strategies to reduce computational costs while ensuring accuracy [39,40]. Surrogate models are also adaptively constructed using deep neural networks in some empirical studies, which are successfully applied to probabilistic failure analysis, significantly reducing the overhead of traditional Monte Carlo methods [41]. Furthermore, conservative surrogate modeling methods are proposed to avoid underestimating risks, maintaining safety margins during the decision-making phase [42]. In the field of materials science, interpretability and visualization analysis are gradually incorporated into the modeling process, ensuring that model outputs can be mutually verified with known laws, thereby enhancing credibility [43]. At the probabilistic level, distribution-free conformal prediction methods are provided as new statistical tools for interval calibration, enabling prediction intervals to be maintained consistent with actual coverage rates [44]. Overall, while hybrid modeling, interpretability tools, and uncertainty calibration have each made progress, the integration of these three elements into an end-to-end framework remains insufficient in concrete compressive strength prediction. When faced with multiple challenges such as small samples, distribution drift, and engineering threshold decisions, the establishment of an integrated surrogate modeling process is particularly necessary.

Existing PIML frameworks predominantly embed governing PDE residuals into the loss function [11,36], yet the mapping from concrete mix proportions to compressive strength is governed by hydration chemistry and time-dependent SCM reactivity rather than a single discretizable PDE. This study therefore adopts a physics-prior residual-learning strategy, in which a domain-specific constitutive baseline captures the dominant physical trends and a compact neural network learns only the bounded residual. Here, the term “residual model” refers to a hybrid architecture in which the neural network is trained to predict the residual (i.e., the difference) between the physics baseline and the observed strength, so that the network corrects only the portion left unexplained by the physics, rather than learning the full input–output mapping from scratch. Component-wise interpretability, three-source uncertainty decomposition and reliability-based design are integrated into a unified framework to support engineering decision-making under uncertainty. In Section 2, a physics-consistent strength baseline is formulated and the overall “physics prior + residual learning” framework with constraints is specified; in Section 3, the dataset and experimental setup are described, the physics-only, data-only, and hybrid models are implemented and comparatively evaluated, SHAP and partial-dependence analyses are performed, uncertainties are decomposed and coverage is calibrated, and reliability design maps over *w*/*b*–*C* are produced; in Section 4, reliability–material trade-offs and the effects of supplementary cementitious material proportions are discussed and engineering implications are distilled; in Section 5, the study is summarized and directions are outlined for stronger physics priors, more robust uncertainty modeling, and multi-objective optimization.

## 2. Methodology

**Fig 1:**
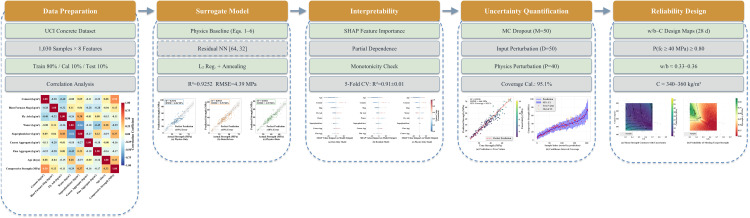
Flowchart of the proposed physics-prior residual-learning framework with calibrated uncertainty for reliability-based concrete mix design.

The overall framework of the proposed methodology is illustrated in Fig 1. The approach proceeds through five stages: (1) data preparation including dataset partitioning and correlation analysis; (2) surrogate model construction combining a physics baseline with a residual neural network under *L*_2_ regularization; (3) interpretability analysis via SHAP feature importance, partial dependence, and monotonicity verification with five-fold cross-validation; (4) uncertainty quantification through MC dropout, input perturbation, physics-parameter perturbation, and post-hoc coverage calibration; and (5) reliability-based design producing *w*/*b*–*C* maps for engineering mix recommendations.

### 2.1. Physics-consistent strength model

A compact baseline consistent with materials physics is formulated. Strength is governed by an effective water-to-binder ratio and by age growth, while time-varying reactivity of supplementary cementitious materials (SCMs) and the superplasticizer-induced reduction of effective water demand are incorporated in a unified manner [45,46]. Let cement, slag, fly ash, mixing water, and superplasticizer dosages be denoted by *C*, *S*, *F*, *W*, and *SP*, respectively; age is denoted by *t*; and compressive strength is denoted by *f*_*c*_. The mapping from mixture proportions to strength is expressed through an effective cementitious content and an effective water content so that the dominant control of the water-to-binder ratio is retained and materials-consistent monotonicity is preserved [47].

The delayed contribution of SCMs is represented by age-dependent reactivity functions that increase monotonically and saturate at long ages,


$$k_{s}(t)=k_{s}\;\frac{t}{\tau_{s}+t},$$ (1a)

$$k_{f}(t)=k_{f}\;\frac{t}{\tau_{f}+t}.$$ (1b)

From (1), an effective cementitious content is defined as


$$C_{eff}(t)=C+k_{s}(t)S+k_{f}(t)F.$$ (2)

The action of superplasticizer is reflected by a normalized, monotone dose–response g(SP)∈[0,1] that reduces the effective water,


$$W_{eff}=W\;[\;1-\eta_{\max}\;g(SP)\;],\;0<\eta_{\max}<1,$$ (3)

and the resulting effective water-to-binder ratio is


$$\omega (t)=\frac{W_{eff}}{C_{eff}(t)}.$$ (4)

Age growth is described either by a power-law relative to a reference age or by a maturity-based function,


$$\phi (t)={\left(\frac{t}{t_{ref}}\right)}^{n},\;n>0,$$ (5a)

ϕ(t)=h(M(t)). (5b)

Combining these ingredients, the baseline strength response is written as


$$f_{c}^{\left(phy\right)}(t)=A{\left(\frac{1}{\omega (t)+\delta }\right)}^{B}\;\phi (t),\;A>0,\;B>0,\;\delta >0.$$ (6)

By construction, a monotone decrease with respect to ω(t) and a monotone increase with respect to *t* are satisfied; SCM later-age gain and superplasticizer–wa*t*er coupling are embedded through *C*_eff_(*t*) and *W*_eff_ [48].

### 2.2. Physics-informed residual coupling model and evaluation

On the basis of the physics-consistent baseline, a residual learning component is introduced. The purpose is to allow the main physics model to capture dominant monotonic relations, while the residual network represents the remaining nonlinear interactions. In this way, the combined model preserves interpretability and improves predictive accuracy [11,49]. To place the physical output and the data-driven residual on the same scale, the target is standardized as


$$y_{i}^{sc}=\frac{y_{i}-\mu_{y}}{\sigma_{y}}$$ (7)

The physics baseline is also mapped to the same scale as


$$y_{i}^{phy,sc}=\frac{f_{c}^{\left(phy\right)}(x_{i})-\mu_{y}}{\sigma_{y}}$$ (8)

The residual network output is denoted by $r(x_{i};\theta )$, and the combined standardized prediction is


$${\tilde{y}}_{i}=r(x_{i};\theta )+y_{i}^{phy,sc}$$ (9)

The training objective is expressed as a sum of the data consistency term and the residual regularization term [50]


$$ℒ=\frac{1}{N}\sum_{i=1}^{N}{\left({\tilde{y}}_{i}-y_{i}^{sc}\right)}^{2}+\lambda_{r}\frac{1}{N}\sum_{i=1}^{N}r(x_{i};\theta {)}^{2}$$ (10)

The prediction is then returned to the original dimension by inverse standardization


$${\hat{y}}_{i}=\sigma_{y}\;{\tilde{y}}_{i}+\mu_{y}$$ (11)

For performance evaluation, several metrics are employed [51]. The coefficient of determination is defined as


$$R^{2}=1-\frac{\sum_{i=1}^{N}(y_{i}-{\hat{y}}_{i}{)}^{2}}{\sum_{i=1}^{N}(y_{i}-\bar{y}{)}^{2}}$$ (12)

The root mean square error measures the average squared deviation


$$RMSE=\sqrt{\frac{1}{N}\sum_{i=1}^{N}(y_{i}-{\hat{y}}_{i}{)}^{2}}$$ (13)

The mean absolute error expresses the mean absolute deviation


$$MAE=\frac{1}{N}\sum_{i=1}^{N}|y_{i}-{\hat{y}}_{i}|$$ (14)

The mean absolute percentage error reflects the relative error level


$$MAPE=\frac{100}{N}\sum_{i=1}^{N}\left|\frac{y_{i}-{\hat{y}}_{i}}{y_{i}+\varepsilon }\right|$$ (15)

Through this formulation, the hybrid model maintains the monotonic relations prescribed by physical knowledge, while the residual learning enhances the flexibility to capture complex behaviors. The evaluation metrics provide a comprehensive assessment of accuracy and robustness.

### 2.3. Interpretive methodology

A physics-informed residual hybrid model is presented in an additive form, where the overall prediction is expressed as the sum of a physics-consistent baseline and a data-driven residual. The baseline is intended to encode dominant relations such as water-to-binder and age effects and to preserve monotonicity and scale consistency, while the residual is intended to capture nonlinear and interaction patterns that are not explicitly represented by the baseline. This design is suitable for diagnosis and presentation without reliance on implementation details.

The overall predictor is written as


$$f(x)=f_{phy}(x)+r(x)\;.$$ (16)

Here, *f*_phy_(*x*) denotes the physics-consistent baseline and *r*(*x*) denotes the incremental data-driven correction. To examine the marginal effect of a single variable, the partial dependence function is introduced


$${PD}_{j}(z)={𝔼}_{X_{-j}}\;[f(z,X_{-j})]\;,$$ (17)

which averages over the complementary features and produces a population-level response curve that facilitates checks of physically reasonable monotonicity and saturation [52]. To describe sample-level heterogeneity, the individual conditional expectation curve is introduced


$${ICE}_{i,j}(z)=f(z,\;x_{i,-j})\;,$$ (18)

which traces the response of a single variable while holding the remaining features of a given sample fixed, thereby revealing background-dependent differences, threshold regions, and local nonlinearities [53]. Under the hybrid setting, PD and ICE can be computed for the overall predictor *f* or applied separately to *f*_phy_ and *r* to distinguish the physics-driven component from the data-driven adjustment and to cross-check their consistency.

For a globally and locally consistent attribution, a Shapley-based additive explanation is adopted to approximate any prediction by a sum of a baseline term and feature attributions


$$f(x)\approx \phi_{0}+\sum_{j=1}^{p}\phi_{j}(x)\;,$$ (19)

where $\phi_{0}=𝔼[f(X)]$ denotes the global baseline and $\phi_{j}(x)$ denotes the marginal contribution of the *j*-th feature at *x*. The conservation property


$$\sum_{j=1}^{p}\phi_{j}(x)=f(x)-𝔼\;\left[f(X)\right]$$ (20)

is used to verify numerical self-consistency of the attribution and to compare the relative importance of different variables across regions [54]. Within the physics-informed residual framework, attributions can be computed either for the overall predictor *f* or split into baseline *f*_phy_ and residual *r*, which enables verification that key monotonic relations are primarily carried by the baseline and quantification of the residual’s incremental contribution to complex nonlinear details. This combination provides explicit support, at the interpretive level, for extrapolation soundness and engineering usability.

### 2.4. Refined uncertainty quantification

A refined uncertainty quantification scheme is presented to provide interval predictions that align nominal confidence with empirical coverage. The approach is designed for a physics-informed residual hybrid so that dominant physical structure is retained by the baseline and remaining variability is attributed to model and data sources in a transparent manner. The use of Monte Carlo dropout for epistemic variability and the use of post-hoc coverage calibration for interval reliability are consistent with established practices in uncertainty quantification and calibrated regression [55–57]. A deterministic baseline on the original scale is defined by an additive composition of a standardized residual output and a standardized physics baseline, followed by an inverse standardization


$${\hat{y}}_{i}^{\left(0\right)}=\sigma_{y}(r(x_{i})+y^{phy}(x_{i}))+\mu_{y}$$ (21)

where *x*_*i*_ denotes the feature vector of mixture and age for the *i*-th instance, r(·) denotes the residual prediction on the standardized scale, $y^{phy}(\cdot )$ denotes the physics baseline on the same scale, and $\mu_{y},\sigma_{y}>0$ denote the mean and standard deviation of the target used for scaling.

Model (epistemic) variability of the residual component is represented by Monte Carlo dropout; stochastic forward passes produce sample predictions [55]


$${\hat{y}}_{i}^{\left(m\right)}=\sigma_{y}(r^{\left(m\right)}(x_{i})+y^{phy}(x_{i}))+\mu_{y},\;m=1,\ldots ,M$$ (22)

where $r^{\left(m\right)}(\cdot )$ denotes the residual with a randomized dropout mask. Under the variational-inference interpretation of Gal and Ghahramani [55], each dropout mask corresponds to a sample from an approximate posterior over the network weights, so the dispersion across *M* forward passes is a principled measure of epistemic uncertainty that reflects insufficient training data or model capacity [58].

Data (aleatory) variability is represented by small zero-mean perturbations in standardized and raw feature spaces to account for measurement and batching fluctuations


$${\hat{y}}_{i}^{\left(d\right)}=\sigma_{y}(r(x_{i}+\varepsilon_{x}^{\left(d\right)})+y^{phy}(x_{i}^{raw}+\varepsilon_{raw}^{\left(d\right)}))+\mu_{y},\;d=1,\ldots ,D$$ (23)

where $\varepsilon_{x}^{\left(d\right)}$ and $\varepsilon_{raw}^{\left(d\right)}$ denote small perturbations in standardized and raw features respectively. Because these perturbations simulate measurement noise and batching variability that would persist even with unlimited training data, the resulting spread is classified as aleatoric uncertainty in the sense of Der Kiureghian and Ditlevsen [58,59].

Parametric epistemic variability of the physics prior is represented by mild perturbations of physics parameters


$${\hat{y}}_{i}^{\left(p\right)}=\sigma_{y}(r(x_{i})+y^{phy}(x_{i};\;\theta +\varepsilon_{\theta }^{\left(p\right)}))+\mu_{y},\;p=1,\ldots ,P$$ (24)

where θ denotes the physics parameter vector and $\varepsilon_{\theta }^{\left(p\right)}$ denotes a bounded perturbation. Because the physics parameters are calibrated on finite training data, they carry estimation uncertainty; perturbing them within bounded ranges and computing the output variance is analogous to a parametric bootstrap around the point estimate, quantifying the parametric component of epistemic uncertainty [38,59].

All sampled predictions are pooled to estimate the predictive mean and dispersion


$$\mu_{i}=\frac{1}{K}\sum_{k=1}^{K}{\hat{y}}_{i}^{\left(k\right)},\;s_{i}=\sqrt{\frac{1}{K-1}\sum_{k=1}^{K}({\hat{y}}_{i}^{\left(k\right)}-\mu_{i}{)}^{2}}$$ (25)

where *K* = *M* + *D* + *P* denotes the total number of retained samples for instance *i*.

A scalar calibration factor is introduced so that nominal and empirical coverage match at a target two-sided level *q* [56]. With the Gaussian quantile *z*_*q*_, the calibrated interval is


$${CI}_{i}^{\left(q\right)}=[\mu_{i}-z_{q}\;\alpha \;s_{i},\;\mu_{i}+z_{q}\;\alpha \;s_{i}]$$ (26)

and α>0 is selected to satisfy


$$\frac{1}{N}\sum_{i=1}^{N}𝕀\{y_{i}\in {CI}_{i}^{\left(q\right)}\}\approx q$$ (27)

so that reported confidence levels are empirically reliable. This post-hoc scalar recalibration follows the framework of Kuleshov et al. [56]: a dedicated calibration partition is used to determine α, and all subsequent coverage statistics are reported on an independent held-out test set that is never used during fitting or calibration, thereby avoiding circularity. Empirically, the single scalar α achieves simultaneous calibration across the 90%, 95%, and 99% confidence levels (Section 3), supporting the approximate validity of this single-parameter recalibration.

Source-wise dispersions are computed from within-mechanism samples to clarify contributions


$$s_{i}^{phy}=std(\{{\hat{y}}_{i}^{\left(p\right)}\}),\;s_{i}^{res}=std(\{{\hat{y}}_{i}^{\left(m\right)}\}),\;s_{i}^{data}=std(\{{\hat{y}}_{i}^{\left(d\right)}\})$$ (28)

and aggregate measures are summarized as


$$s_{i}^{epi}=\sqrt{(s_{i}^{phy}{)}^{2}+(s_{i}^{res}{)}^{2}},\;s_{i}^{tot}=\alpha \;s_{i}$$ (29)

where $s_{i}^{epi}$ denotes epistemic (model) uncertainty and $s_{i}^{tot}$ denotes the calibrated total uncertainty. In this refined scheme, model variability is represented by dropout sampling, data variability is represented by input perturbations, physics-prior variability is represented by parameter perturbations, and calibration aligns nominal and empirical coverage for engineering use [57].

## 3. Case study

### 3.1. Data description

This study employs the public dataset “Concrete Compressive Strength Data Set” collected by Yeh et al., which was obtained from high-performance concrete mix experiments and is available in the UCI Machine Learning Repository. The dataset comprises 1,030 samples with nine variables: eight input features (cement, blast furnace slag, fly ash, water, superplasticizer, coarse aggregate, fine aggregate, and age) and one output variable (compressive strength). Each variable is measured in kg/m³ or days, with compressive strength expressed in MPa. As the data source is reliable, publicly transparent, and widely used in concrete performance research, it provides a credible foundation for subsequent machine learning model training. Although this dataset has been extensively employed as a benchmark in concrete strength research, its adoption here is deliberate: the primary contribution of this study is a methodological framework integrating physics priors, residual learning, interpretability, and calibrated uncertainty, which is best evaluated on a well-characterised, publicly reproducible benchmark that permits fair comparison with existing methods. To mitigate concerns regarding over-reliance on a single data partition, five-fold cross-validation is additionally performed and reported in Section 3.2.

The descriptive statistics (see Table 1) reveal significant variations in the ranges and distribution characteristics of different raw materials. Cement content ranges from 102.00 to 540.00 kg/m³ with a mean of 281.17 kg/m³ and standard deviation of 104.51 kg/m³, indicating substantial mix design diversity. Water content shows a narrower range from 121.80 to 247.00 kg/m³ with a mean of 181.57 kg/m³ and relatively small standard deviation of 21.35 kg/m³, suggesting more consistent water usage. Blast furnace slag and fly ash both have minimum values of 0, with means of 73.90 and 54.19 kg/m³ respectively, and high standard deviations (86.28 and 64.00 kg/m³), indicating these supplementary materials are optional in many mix designs. Superplasticizer usage is minimal with a mean of only 6.20 kg/m³. Age data exhibits extreme variability, ranging from 1 to 365 days with a mean of 45.66 days and standard deviation of 63.17 days. The compressive strength varies from 2.33 to 82.60 MPa with a mean of 35.82 MPa and standard deviation of 16.71 MPa. Addressing these characteristics through min-max normalization and outlier screening before modeling can significantly improve training stability. Skewness and excess kurtosis are also reported in Table 1 to characterise distributional shape. Most mixture variables exhibit mild positive skewness (0.07–0.91), while age stands out with a skewness of 3.27 and an excess kurtosis of 12.17, indicating a strongly right-skewed and heavy-tailed distribution dominated by short-age specimens. Fly ash shows a negative excess kurtosis of -1.33, reflecting a platykurtic (flat) distribution due to the large number of zero-dosage samples. These observations motivate the standardization and robust training strategies adopted in this study.

**Table 1:** Descriptive statistics of concrete mixture components showing range and variability characteristics.

Variable	Min	Max	Mean	Std	Skewness	Kurtosis
Cement (kg/m³)	102.00	540.00	281.17	104.51	0.51	−0.52
Blast Furnace Slag (kg/m³)	0.00	359.40	73.90	86.28	0.80	−0.51
Fly Ash (kg/m³)	0.00	200.10	54.19	64.00	0.54	−1.33
Water (kg/m³)	121.80	247.00	181.57	21.35	0.07	0.12
Superplasticizer (kg/m³)	0.00	32.20	6.20	5.97	0.91	1.41
Coarse Aggregate (kg/m³)	801.00	1145.00	972.92	77.75	−0.04	−0.60
Fine Aggregate (kg/m³)	594.00	992.60	773.58	80.18	−0.25	−0.10
Age (days)	1.00	365.00	45.66	63.17	3.27	12.17
Compressive Strength (MPa)	2.33	82.60	35.82	16.71	0.42	−0.31

**Fig 2:**
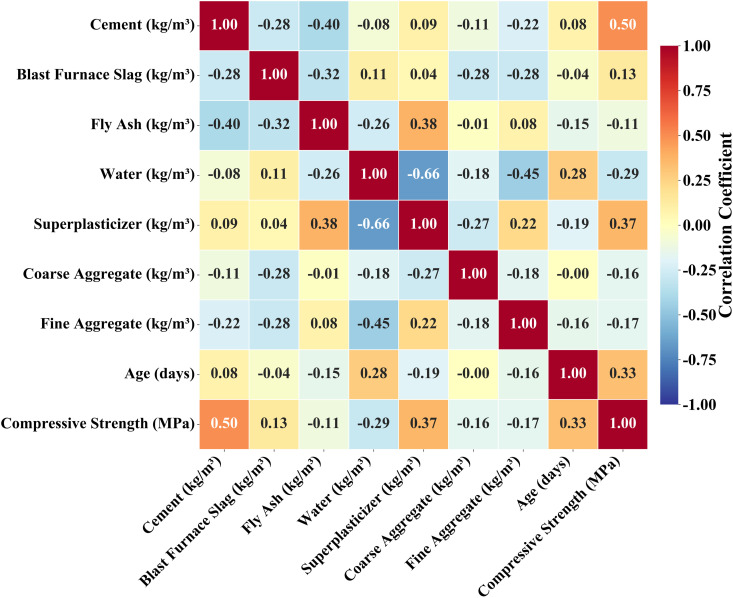
Correlation coefficient heatmap for concrete mixture components and compressive strength relationships.

The correlation analysis results are presented in Fig 2. Cement content (correlation coefficient approximately 0.50), superplasticizer (0.37), and age (0.33) show positive correlations with compressive strength. Conversely, water, coarse aggregate, and fine aggregate exhibit negative correlations with compressive strength, with water showing the strongest negative correlation (−0.29). Blast furnace slag has a correlation coefficient of 0.13, indicating minimal impact on strength. These statistical relationships can identify key factors influencing strength, guide feature weight settings in the model, and through embedding material mechanics principles as physical constraints, help improve the generalization capability of predictive models.

### 3.2. Surrogate model development

In order to keep the scale and the main rules of the physical baseline consistent with the data, a global calibration of the physical model defined by equations (1), (3), (4), and (6) is carried out on the training set. An 80/20 split and the mean squared error criterion are used, and differential evolution within bounded domains is applied. Several restarts are performed to ensure stable convergence. The optimal parameters are fixed and used for all experiments. With this parameter set, the “Physics-Only” baseline shows on the independent test set *R*^2^ = 0.8176, RMSE = 6.86 MPa, and MAE = 5.57 MPa. These results indicate that the physical prior captures the main effects of water-to-binder ratio and age and provides a consistent and reproducible base. A small residual network is then added on top of the baseline to correct the remaining deviations, so that accuracy and robustness are improved without loss of physical consistency. The complete set of baseline parameters, including their physical meanings, units, types and calibrated values, is summarised in Table 2. The fitted exponent *B* = 2.23 is consistent with classical Abrams’ law, which typically yields exponents between 1.5 and 3.0 for ordinary Portland cement systems. The preset constants $\tau_{s}=7$ days and $\tau_{f}=14$ days reflect the well-documented observation that slag hydration proceeds faster than fly-ash pozzolanic reaction, and $t_{ref}=28$ days corresponds to the standard curing age adopted in concrete engineering practice.

**Table 2:** Physics baseline parameters: physical meaning, unit, type and calibrated value.

Parameter	Physical meaning	Unit	Type	Bounds	Value
*A*	Strength scaling coefficient	MPa	Fitted	[20,60]	34.56
*B*	*w*/*b* sensitivity exponent	—	Fitted	[1.5, 2.5]	2.23
*k*_*s*_	Ultimate slag reactivity	—	Fitted	[0.5, 1.2]	1.05
*k*_*f*_	Ultimate fly-ash reactivity	—	Fitted	[0.3, 0.8]	0.80
$\eta_{\max}$	Max SP water-reduction ratio	—	Fitted	[0.1, 0.35]	0.10
*n*	Age power-law exponent	—	Fitted	[0.15, 0.35]	0.18
δ	Denominator regulariser	—	Preset	—	0.5
$\tau_{s}$	Slag half-saturation age	day	Preset	—	7.0
$\tau_{f}$	Fly-ash half-saturation age	day	Preset	—	14.0
$t_{ref}$	Reference curing age	day	Preset	—	28.0

In the surrogate model development, three modeling strategies are constructed and compared: a physics-based model (Physics-Only), a purely data-driven model (Data-Driven), and a residual-coupled hybrid model (Physics+Data). The Physics-Only model is established on effective water–cement ratio and age-dependent growth, where time-varying contributions of supplementary cementitious materials and the reduction of effective water by superplasticizer are incorporated, and the compressive strength is directly calculated. The Data-Driven model is designed as a fully connected neural network with two hidden layers (ReLU activation, dropout regularization, Adam optimizer, and early stopping), using eight standardized mixture features as inputs. The Physics+Data model adopts a framework of “physics prior + neural network residual learning”: a baseline prediction is obtained from the physics model, and the neural network learns the residuals with an *L*_2_ regularization term (annealed along epochs), thereby balancing interpretability and predictive capability. The dataset is split into training and testing subsets, all features and targets are standardized, and robustness tests are performed with 40%–100% fractions of the training data to evaluate generalization under limited-sample scenarios. For subsequent uncertainty quantification, a three-way split is adopted: 80% of the samples are used for model training, 10% for calibration of the uncertainty scaling factor α, and 10% as a held-out test set on which all reported coverage statistics are evaluated, ensuring that the calibration is validated on genuinely unseen data [56].

**Fig 3:**
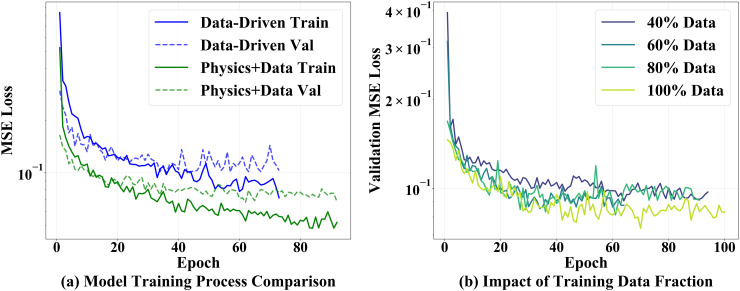
(a) Training process comparison between Data-Driven and Physics+Data models; (b) Impact of training data fraction on validation loss.

**Fig 4:**
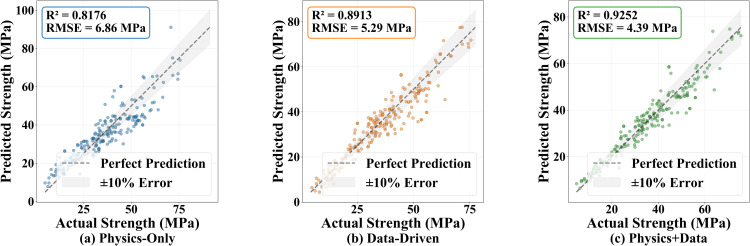
Parity plots comparing predicted and actual compressive strengths for Physics-Only, Data-Driven, and Physics+Data models. The dashed line indicates perfect prediction, and shaded areas represent ±10% error.

As shown in Fig 3(a), the training and validation losses of the Physics+Data model are consistently lower than those of the Data-Driven model, and faster as well as smoother convergence is observed on the logarithmic scale. Fig 3(b) shows that when the fraction of training data increases from 40% to 100%, the validation MSE of Physics+Data continuously decreases. Even with only 40% of the training data, the Physics+Data model achieves a validation error comparable to that obtained with larger datasets, indicating that the physics prior significantly mitigates uncertainty and overfitting risks in small-sample cases. The parity plots of predicted versus actual values in Fig 4 further validate this conclusion: the data points from all three models distribute around the “perfect prediction line,” but those of Physics+Data are the most concentrated, with the highest proportion falling within the ±10% error band and showing the best linear consistency (highest *R*^2^ and lowest RMSE).

The quantitative metrics are summarized in Table 3. Compared with Physics-Only, the Data-Driven model already improves prediction accuracy significantly; however, Physics+Data further enhances the performance, with *R*^2^ reaching 0.9252 and RMSE reduced to 4.39 MPa, while MAE and MAPE also achieve the lowest values. These results indicate that, in the task of predicting concrete compressive strength, embedding interpretable physical principles as a strong prior with residual learning by a small neural network allows the model to maintain physical consistency while achieving higher predictive accuracy and stronger robustness under small-sample conditions. This conclusion is consistent with the faster convergence and more compact error distribution shown in Figs 3 and 4.

**Table 3:** Performance comparison of surrogate models.

Model	*R* ^2^	RMSE (MPa)	MAE (MPa)	MAPE (%)
Physics-Only	0.8176	6.86	5.57	19.05
Data-Driven	0.8913	5.29	3.99	12.49
Physics+Data	0.9252	4.39	3.27	10.46

To verify that the above ranking is not an artefact of the particular 80/20 random split, a five-fold cross-validation (CV) is performed. The full dataset is divided into five disjoint subsets; in each fold four subsets are used for training (with an internal 90/10 sub-split to provide a validation set for early stopping) and the remaining subset serves as the held-out test set. Table 4 reports the mean and standard deviation of each metric across the five folds. The Physics+Data model achieves a mean *R*^2^ of 0.9122 ± 0.0145 and a mean RMSE of 4.91 ± 0.22 MPa, consistently outperforming both alternatives across all folds. The low standard deviations confirm that the hybrid model’s advantage is robust to resampling rather than dependent on a favourable data split.

**Table 4:** Five-fold cross-validation results (mean ± std).

Model	*R* ^2^	RMSE (MPa)	MAE (MPa)	MAPE (%)
Physics-Only	0.8148 ± 0.0082	7.17 ± 0.50	5.55 ± 0.39	19.71 ± 0.75
Data-Driven	0.9002 ± 0.0109	5.25 ± 0.34	3.73 ± 0.17	12.90 ± 1.52
Physics+Data	0.9122 ± 0.0145	4.91±0.22	3.46 ± 0.26	11.83 ± 1.32

### 3.3. Model interpretability analysis

**Fig 5:**
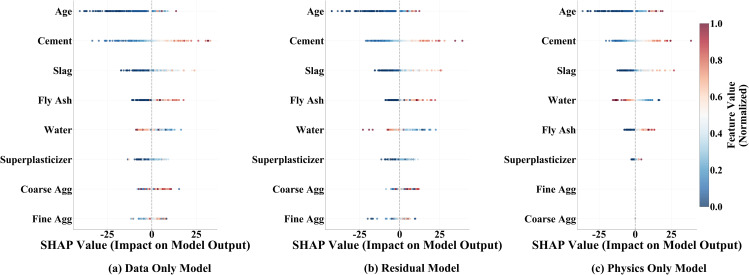
SHAP feature importance for physics-only, data-only and residual models.

To evaluate both interpretability and predictive performance of the hybrid model, a SHAP feature importance analysis is conducted. SHAP values are computed using the entire test set to approximate the contribution of each feature relative to a baseline prediction. In the resulting plot (Fig 5), “age” and “cement” are consistently identified as the most influential factors, with contributions significantly exceeding those of other variables. Moderate positive contributions are assigned to slag and fly ash, whereas water exerts a negative influence, reflecting the physical principle that increased water content weakens concrete. Contributions from superplasticizer, coarse aggregate and fine aggregate are markedly smaller, indicating limited direct impact. The ranking of feature importance in the residual model aligns with material science theory and serves to correct potential artefacts present in purely data-driven approaches, thereby highlighting the hybrid model’s capacity to preserve physically meaningful relationships while mitigating overfitting to noise.

**Fig 6:**
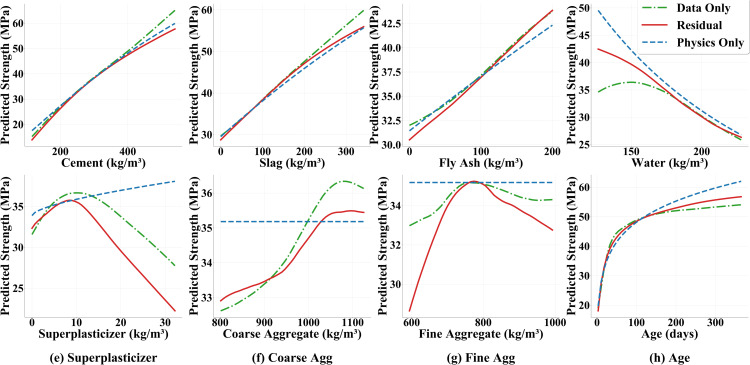
Partial dependence analysis for eight input variables using three models.

To further examine how individual input variables influence the predicted compressive strength, a partial dependence analysis is undertaken. In these plots (Fig 6), each feature is varied across its observed range while all other features are held at their mean values, and the corresponding changes in predicted strength are recorded. The residual model exhibits monotonic increases in predicted strength with increasing cement, slag, fly ash and age, mirroring the physics model’s expectations. A monotonic decrease is maintained for water content, which avoids the spurious oscillations occasionally produced by purely data-driven models. Nonlinear effects are captured for superplasticizer, coarse aggregate and fine aggregate: initial increases in dosage yield higher strength, but further increases lead to a decline, with a peak strength occurring at a superplasticizer dosage around 8–10 kg m^–3^. Throughout these curves, the residual model’s predictions generally lie between those of the physics-only and data-driven models, blending the physically grounded monotonic behaviour with data-informed adjustments. This “physics prior plus data compensation” approach maintains the plausibility of the model’s responses to changes in mixture components, while providing flexibility to capture the complex nonlinearity inherent in the dataset.

### 3.4. Uncertainty analyses

In this uncertainty analysis, the previously trained refined residual PINN model is used. The model combines a physics‐based component based on the water–cement ratio and a residual neural network with two hidden layers (64 and 32 neurons) and ReLU activations. Eight mixture features—cement, slag, fly ash, water, superplasticizer, coarse aggregate, fine aggregate, and age—are input to the model. Gentle MC Dropout is employed, reducing the original dropout rate of 0.1 to 0.03, to sample epistemic uncertainty; Gaussian noise with standard deviations of 0.02 in the standardized feature space and 0.01 in the raw space is added to capture aleatory uncertainty; and physical‐model parameters are perturbed by ±5% (bounded within ±20%) to estimate parameter uncertainty. Multiple predictions are generated for each type of perturbation, and those with *R*^2^ > 0.8 are retained. The predictions are aggregated to compute the mean and standard deviation, and the latter is scaled via binary search so that the 95% confidence interval matches the target coverage. On the test set, the deterministic predictions achieve $R^{2}\approx 0.9158$ and an RMSE of approximately 4.66 MPa.

**Fig 7:**
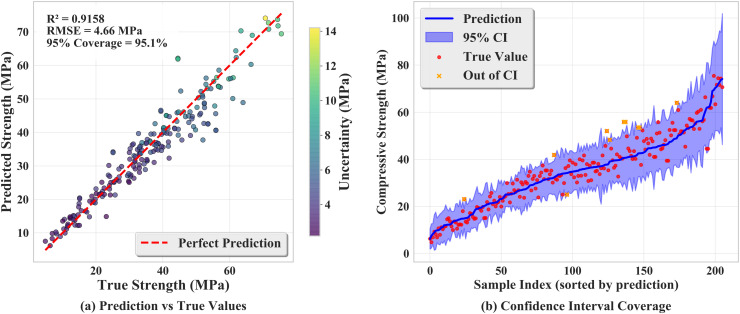
Prediction versus true values and confidence interval coverage.

Fig 7 illustrates the predictive performance and confidence interval coverage. In Fig 7(a), predicted compressive strengths are plotted against true values, and each point is color‐coded by the predicted standard deviation, with darker colors corresponding to lower uncertainty and lighter colors to higher uncertainty. A dashed red line represents perfect predictions, and a strong linear relationship is observed, indicating good predictive accuracy. In Fig 7(b), the test samples are sorted by predicted strength and plotted; the blue band denotes the calibrated 95% confidence interval, red dots correspond to true values, and yellow crosses highlight samples outside the interval. It can be seen that the calibrated confidence band encloses nearly all of the true strengths, and the overall coverage is approximately 95.1%. These visualizations demonstrate that mild Dropout sampling and subsequent calibration yield confidence intervals that align well with the empirical error distribution, while maintaining high predictive accuracy.

**Fig 8:**
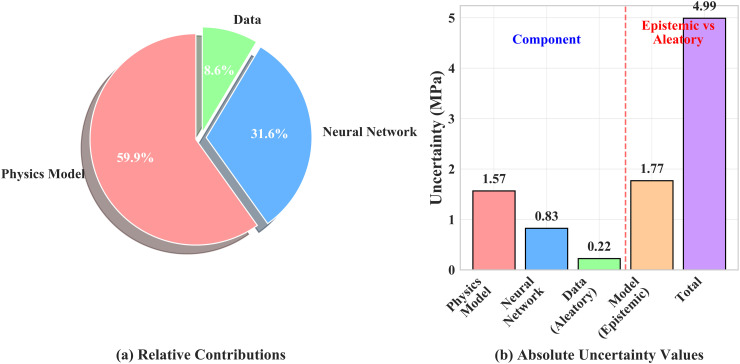
Relative contributions and absolute uncertainty values.

Uncertainty decomposition is visualized in Fig 8. The pie chart in Fig 8a shows the relative contributions of different sources: the physical‐model uncertainty contributes 59.9%, the residual neural‐network uncertainty contributes 31.6%, and the random data noise contributes 8.6%. Fig 8 (b) presents the absolute uncertainty values in MPa. The bars show that the physical‐model uncertainty is 1.57 MPa, the neural‐network uncertainty is 0.83 MPa, and the data noise is 0.22 MPa; a separate bar labeled “Model (Epistemic)” represents the combined epistemic uncertainty of the physical and neural components, calculated to be 1.77 MPa, while the total uncertainty is 4.99 MPa. These results indicate that the overall predictive uncertainty is primarily contributed by the physical model, with smaller contributions from the neural network and data noise. To verify the sufficiency of the adopted sample sizes, convergence diagnostics are performed for each perturbation source. For MC dropout, the relative change in the mean predicted standard deviation is less than 2.5% when the sample count increases from *M* = 30 to *M* = 50 (0.84→0.84 MPa). For physics-parameter perturbation, the relative change from *P* = 30 to *P* = 40 is also less than 2.5% (1.99→1.95 MPa). These results confirm that the current sample sizes (*M* = 50, *P* = 40) have converged and that further increasing the number of draws would not materially alter the uncertainty estimates.

**Fig 9:**
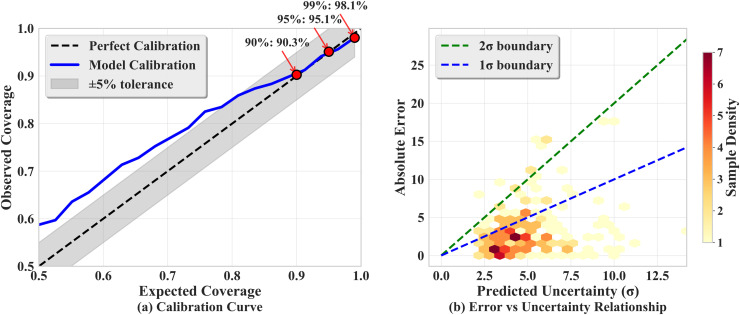
Coverage curve and error versus predicted uncertainty relationship.

Calibration performance and the relationship between error and predicted uncertainty are shown in Fig 9. Fig 9(a) plots the observed coverage against the expected coverage for confidence levels ranging from 0.5 to 0.99. The dashed black line denotes perfect calibration, the blue line shows the model calibration, and the grey band represents a ±5% tolerance. It is evident that the model calibration curve closely follows the ideal line within the tolerance band, with observed coverages of 90.3%, 95.1%, and 98.1% at the 90%, 95%, and 99% confidence levels, respectively. Fig 9 (b) shows a hexbin plot of absolute error versus predicted standard deviation; a positive trend is evident, meaning that larger predicted uncertainties correspond to larger absolute errors. Most samples are located between the 1σ (blue dashed) and 2σ (green dashed) boundaries, indicating that the predicted standard deviation is a reasonable indicator of the true prediction error. To quantify calibration quality, the Expected Calibration Error (ECE) and Maximum Calibration Error (MCE) are computed over ten confidence levels uniformly spaced on [0.50, 0.99]. The overall ECE is 0.0306 at the 95% level and 0.1181 across all bins; the MCE is 0.1699, occurring at the 50% level where the model is slightly conservative (observed coverage exceeds nominal coverage). In the engineering-critical high-confidence region (90%–99%), calibration errors are all below 0.07, indicating that the uncertainty estimates are reliable for risk-based decision-making. Overall, the refined PINN‐based uncertainty analysis yields accurate predictions, reliable confidence intervals, and a clear decomposition of uncertainty sources, while ensuring that the coverage of the confidence intervals is well calibrated.

### 3.5. Reliability-based engineering design maps

A two–dimensional engineering map over the water–to–binder ratio (*w*/*b*) and cement content (*C*) is constructed on top of the trained residual hybrid surrogate (Physics+Data). The design grid is defined as w/b∈[0.30,0.70] and $C\in [150,\;500]\;kg\;m^{-3}$ at 28 days; slag and fly-ash mass fractions are fixed at 15% and 10% of the binder, respectively, implying b=C/(1−0.15−0.10). The superplasticizer dosage is set to $6\;kg\;m^{-3}$, and the coarse/fine aggregates are fixed at $973/774\;kg\;m^{-3}$. The physics sub-model follows an “effective water–binder ratio + age-factor” formulation that accounts for water reduction by the superplasticizer and the time-dependent contributions of supplementary cementitious materials; the residual neural network supplies data-driven corrections to the physics baseline. Predictive uncertainty is estimated by gentle Monte-Carlo dropout with *T* = 40 forward passes (dropout scaling 0.3) and by superposing *K* = 10 draws of 5% Gaussian perturbations to the physics parameters, clipped within ±20%. The resulting standard deviation is then multiplied by the previously obtained scale factor to achieve the 95% target coverage, and the probability of meeting the 40 MPa target is computed as $P=\overset{―}{\Phi }\;((40-\mu )/\sigma )$. A fixed random seed is adopted to ensure reproducibility, and observational mixes from the dataset are overlaid to indicate data coverage.

**Fig 10:**
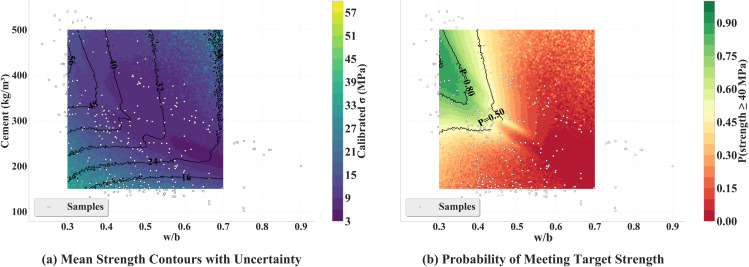
Design map composed of two panels: (a) mean-strength isolines overlaid on the calibrated standard deviation σ; (b) reliability for the 40 MPa target.

In Fig 10, the left panel presents the calibrated σ field with overlaid mean-strength contours. The contours display an almost vertical orientation with respect to the cement axis over the interior of the domain (e.g., 16, 24, 32, 40, 48 MPa), indicating that strength is predominantly governed by *w*/*b*, whereas the sensitivity to *C* at fixed *w*/*b* is weaker. Increased σ values are observed near the edges of the design space, where extrapolation is expected. The right panel shows the reliability map for the 40 MPa target; only the contour levels actually obtained in the computation are displayed, namely *P* = 0.50 and *P* = 0.80. Regions with *P* ≥ 0.80 are confined to relatively low *w*/*b* (approximately ≲0.33−0.35) combined with moderate-to-high cement contents, and the probability decreases rapidly as *w*/*b* increases, which is consistent with the physical control of strength by the water–binder ratio.

**Fig 11:**
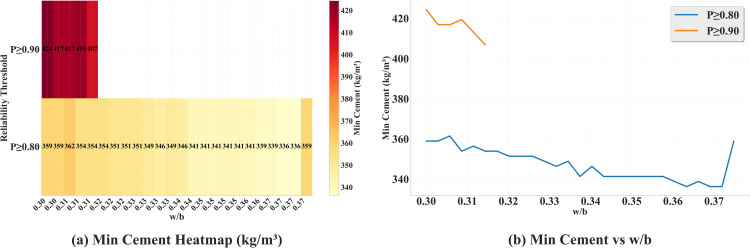
Reliability-conditioned recommendations derived from Fig 10: (a) heatmap of the minimum cement content (kg/m^3^) required at each *w*/*b* to satisfy a given reliability threshold; (b) the corresponding “minimum cement vs *w*/*b*” curves for *P* ≥ 0.80 and *P* ≥ 0.90.

Fig 11 visualizes the recommendation table implied by the reliability map. For the stricter requirement *P* ≥ 0.90, feasible solutions are found only up to w/b≲0.32, and the minimum cement demand is approximately $407-424\;kg\;m^{-3}$. When the threshold is relaxed to *P* ≥ 0.80, the feasible range expands to w/b≈0.30−0.38, and the minimum cement content is reduced to about $336-359\;kg\;m^{-3}$; a relatively flat segment appears around w/b≈0.33−0.37, which is suitable for cost–reliability trade-offs. In practice, the region w/b≈0.33−0.36 with $C\approx 340-360\;kg\;m^{-3}$ may therefore be selected to attain *P* ≥ 0.80, whereas achieving *P* ≥ 0.90 requires restricting w/b≲0.32 and raising *C* to about $410\;kg\;m^{-3}$ or above; attention to quality control is warranted in these low-*w*/*b* zones where the uncertainty field is larger. These recommendations are conditioned on the fixed proportions (slag 15%, fly ash 10%, superplasticizer $6\;kg\;m^{-3}$) and the 28-day age assumed in the maps, and are consistent with the modeling and uncertainty-calibration protocol adopted in this work. To provide an empirical check on the surrogate-derived reliability regions, Table 5 compares the model-based recommendations with the observed 28-day compressive strengths in the dataset. Among the 45 observed mixtures falling within the recommended zone (w/b∈[0.30,0.38] and $C\geq 300\;kg\;m^{-3}$), the empirical exceedance rate $\hat{P}(f_{c}\geq 40\;MPa)$ is 97.8% (95% Wilson CI: [0.884, 0.996]), which is consistent with the surrogate prediction of *P* ≥ 0.80. By contrast, for mixtures with *w*/*b* > 0.40 the empirical exceedance rate drops to 11.6% (95% Wilson CI: [0.084, 0.159]). This monotone decline of the observed exceedance rate across *w*/*b* bands mirrors the contour pattern of the model-based reliability map and supports the physical plausibility of the recommended design window. The recommended *w*/*b* range of 0.33–0.36 is also consistent with ACI 211 guidance for target strengths around 40 MPa [60].

**Table 5:** Empirical validation of reliability regions using observed 28-day strengths.

*w*/*b* range & *C* filter	*n*	Mean (MPa)	Std (MPa)	$\hat{P}(\geq 40)$	95% Wilson CI
≤0.33, *C* ≥ 300	57	58.7	11.1	96.5%	[0.881, 0.990]
0.33–0.36, *C* ≥ 300	12	57.4	7.8	91.7%	[0.646, 0.985]
0.36–0.40, *C* ≥ 300	26	47.5	8.9	88.5%	[0.710, 0.960]
> 0.40 (all *C*)	284	29.5	9.3	11.6%	[0.084, 0.159]

## 4. Discussion

**Fig 12:**
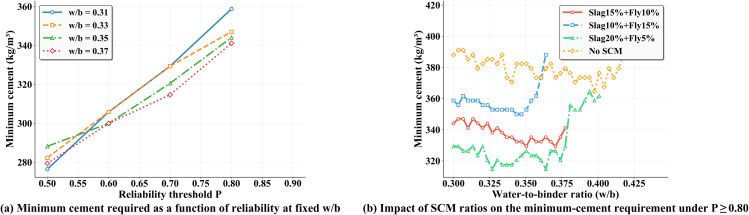
Reliability–material trade-offs and SCM composition effects at 28 days for the 40 MPa target.

The same Physics+Data residual surrogate and the calibrated uncertainty procedure described earlier are used to build the two-dimensional design maps at 28 days over w/b∈[0.30,0.70] and $C\in [150,500]\;{\text{kg m}}^{-3}$ with default SCMs (slag 15% + fly ash 10%), superplasticizer $6\;{\text{kg m}}^{-3}$, and coarse/fine aggregate $973/774\;{\text{kg m}}^{-3}$. On this basis, minimum-cement–versus–reliability curves are obtained at representative w/b∈{0.31,0.33,0.35,0.37} by scanning cement content under the constraint $P\geq P_{thr}$ (Fig 12(a)). A near linear–to–convex growth in the required cement is observed when *P*_thr_ rises from 0.50 to 0.80, and the low-*w*/*b* curves increase faster at the high-*P* end. This behavior is not in conflict with the higher mean strength at low *w*/*b*, but is explained by larger σ in that region; under a reliability constraint, the lower bound μ−1.96σ becomes harder to exceed the 40 MPa target and extra cement is demanded. These results indicate that reliability-driven mix optimization should account for uncertainty explicitly instead of using the mean strength alone.

At the same reliability level, the default SCM (slag 15% + fly ash 10%) is compared with three variants: slag 10% + fly ash 15%, slag 20% + fly ash 5%, and no SCM. The curves of “minimum cement versus *w*/*b*” under *P* ≥ 0.80 are shown in Fig 12(b). It is observed that *slag 20% + fly ash 5%* requires the least cement, followed by the default *slag 15% + fly ash 10%*; *slag 10% + fly ash 15%* and *no SCM* show medium and highest demands, respectively. This ranking is consistent with the time-dependent reactivity in the physics prior at 28 days, where the effective contribution of slag exceeds that of fly ash, so a higher slag fraction reduces the cement required to satisfy *P* ≥ 0.80 at fixed *w*/*b*. When w/b>rsim0.38, all curves rise sharply, reflecting the combined effect of decreasing mean strength and increasing σ; therefore the reliability cost grows rapidly in that zone. A practical trade-off region that balances economy, constructability, and reliability is indicated around w/b≈0.33−0.37, where the *slag-rich* schemes yield flatter profiles and are easier to implement with stable reliability.

When reliability is used as a constraint or a target, the optimal *w*/*b* is not always the smallest value; the uncertainty penalty can move the optimum toward the mid-*w*/*b* range. At 28 days and for the 40 MPa target, increasing the slag fraction and decreasing the fly-ash fraction lowers the cement needed to reach *P* ≥ 0.80. These conclusions are conditioned on the present setting (age 28 days, target 40 MPa, fixed SCM mass fractions, and default admixture/aggregate levels). If the age, the target strength, or the reliability threshold changes, the recommended region will shift accordingly. All analyses remain consistent with the Physics+Data residual framework and the calibrated uncertainty procedure used in the previous sections.

It is instructive to compare the reliability-based approach with the traditional overdesign method commonly used in concrete practice. In the conventional approach, a required average strength $f_{cr}^{\prime }$ is computed from the specified characteristic strength $f_{c}^{\prime }$ and a site-level standard deviation *s* using fixed formulas (e.g., $f_{cr}^{\prime }=f_{c}^{\prime }+1.34s$ or $f_{c}^{\prime }+2.33s-3.45$, whichever is larger). For $f_{c}^{\prime }=40$ MPa and *s* = 4 MPa, this yields $f_{cr}^{\prime }\approx 45.9$ MPa, i.e., a uniform overdesign of 14.7% that applies identically across the entire design space regardless of actual local uncertainty. In contrast, the present reliability-based framework adapts the safety margin spatially through the calibrated σ field (Fig 10a): in well-covered mid-*w*/*b* regions where σ≈3 MPa, achieving *P* ≥ 0.80 requires only ≈6.3% overdesign above the target; in low-*w*/*b* boundary regions where σ≈8 MPa, the overdesign automatically increases to ≈16.8%. This spatial adaptivity offers a twofold advantage: cement is saved in well-understood regions while greater safety margins are automatically imposed in high-uncertainty zones, thereby reconciling economy and structural safety in a principled manner.

From a practical standpoint, the proposed framework can be adapted to realistic mix optimization under material variability. The physics-prior sub-model explicitly parameterises the time-dependent reactivities of slag and fly ash (*k*_*s*_, *k*_*f*_) and the superplasticizer efficiency ($\eta_{\max}$), so that reliability maps can be regenerated for different SCM proportions with minimal effort, as demonstrated in Fig 12(b). When a concrete batching plant switches raw-material sources, only the six physics parameters need to be recalibrated via differential evolution on a small set of trial-mix results; the residual network architecture, the uncertainty decomposition pipeline, and the post-hoc coverage calibration remain unchanged. This modular design provides a practical pathway for generating project-specific reliability maps from limited calibration data, replacing extensive trial batching with model-assisted decision-making under quantified uncertainty.

## 5. Conclusions and future works

This study is based on the publicly available UCI Concrete Compressive Strength dataset, where three types of surrogate models are systematically constructed and compared: a Physics-Only model based on effective water-to-binder ratio and age effects, a Data-Driven model using a two-layer fully connected network, and a Physics+Data hybrid model combining physical priors with residual learning. Experimental results demonstrate that the Physics+Data model achieves *R*^2^ = 0.9252 and *RMSE* = 4.39 MPa (MAE = 3.27 MPa, MAPE = 10.46%) on an independent test set, showing significant improvements over both Physics-Only and Data-Driven approaches. These gains are confirmed by five-fold cross-validation with low inter-fold variance (Table 4). When only 40% of training data is used, validation errors remain close to those obtained with full data, indicating robustness to small samples and faster, more stable convergence. Interpretability analysis further reveals that SHAP values and partial dependence plots consistently identify the physical laws of “positive contributions from age and cementitious materials (especially cement) and negative contribution from water content.” The hybrid model maintains monotonicity of key variables while capturing nonlinear marginal effects of superplasticizers and aggregates, balancing physical interpretability with data fitting capability.

For uncertainty quantification, mild MC-Dropout, feature and label perturbation, and joint sampling of physical parameters within ±5% (capped at ±20%) are employed. Calibrated confidence intervals are obtained through coverage calibration: the actual coverage rate of 95% confidence bands on the test set is approximately 95.1%, with point estimation performance maintained at $R^{2}\approx 0.9158$ and RMSE≈4.66 MPa. Uncertainty decomposition shows that total uncertainty is dominated by contributions from the physical sub-model (approximately 59.9%), followed by the neural network (31.6%), with data noise being relatively small (8.6%). This suggests that future efforts should focus on reducing uncertainty in physical prior parameters. Reliability mapping based on calibrated uncertainty reveals that compressive strength at 28 days is primarily controlled by *w*/*b* ratio. Low *w*/*b* regions exhibit high mean strength but also high variance. When targeting 40 MPa with *P* ≥ 0.80, recommendations include w/b≈0.33−0.36 and C≈340−360 kg m^−3^. For enhanced reliability with *P* ≥ 0.90, w/b≲0.32 and C>rsim410 kg m^−3^ are required. At 28 days, slag-rich and fly ash-lean mixtures are more favorable for reducing cement content while achieving given reliability levels, though higher uncertainty penalties and construction workability concerns should be noted at design domain boundaries and low *w*/*b* regions.

Future work will focus on stronger physical priors, more robust uncertainty modeling, and multi-objective optimization for engineering decisions. First, physical mechanisms including age evolution based on hydration kinetics and thermal/moisture curing conditions, admixture-binder ratio coupling, and aggregate moisture states will be incorporated at the physical level, with assumption space further constrained through monotonicity/shape constraints and piecewise differentiable priors. Second, explicit heteroscedastic and heavy-tailed error models and hierarchical Bayesian approaches will be introduced at the statistical level to absorb mixture-plant-batch hierarchical differences, combined with out-of-distribution detection and domain adaptation to improve cross-data source generalization. Third, at the decision level, cost and embodied CO_2_, workability/pumpability indicators will be jointly incorporated with strength-reliability into robust/risk-averse optimization, with active learning and optimal experimental design guiding incremental experiments to maximize information gain. Finally, external validation on broader age ranges and target strengths, different SCM combinations, and real engineering field data is recommended, with extension to durability indicators (such as permeability and chloride ion migration resistance) to establish a reusable and transferable digital twin framework for mixture design.
